# Auxiliary activation of the complement system and its importance for the pathophysiology of clinical conditions

**DOI:** 10.1007/s00281-017-0646-9

**Published:** 2017-09-12

**Authors:** Markus Huber-Lang, Kristina N. Ekdahl, Rebecca Wiegner, Karin Fromell, Bo Nilsson

**Affiliations:** 1 Institute of Clinical and Experimental Trauma-Immunology, University Hospital Ulm, 89081 Ulm, Germany; 2 Department of Immunology, Genetics and Pathology (IGP), Rudbeck Laboratory C5:3, Uppsala University, SE-751 85 Uppsala, Sweden; 3 Linnæus Center of Biomaterials Chemistry, Linnæus University, SE-391 82 Kalmar, Sweden

**Keywords:** Complement system, Proteases, Protease inhibitors, Trauma

## Abstract

Activation and regulation of the cascade systems of the blood (the complement system, the coagulation/contact activation/kallikrein system, and the fibrinolytic system) occurs via activation of zymogen molecules to specific active proteolytic enzymes. Despite the fact that the generated proteases are all present together in the blood, under physiological conditions, the activity of the generated proteases is controlled by endogenous protease inhibitors. Consequently, there is remarkable little crosstalk between the different systems in the fluid phase. This concept review article aims at identifying and describing conditions where the strict system-related control is circumvented. These include clinical settings where massive amounts of proteolytic enzymes are released from tissues, e.g., during pancreatitis or post-traumatic tissue damage, resulting in consumption of the natural substrates of the specific proteases and the available protease inhibitor. Another example of cascade system dysregulation is disseminated intravascular coagulation, with canonical activation of all cascade systems of the blood, also leading to specific substrate and protease inhibitor elimination. The present review explains basic concepts in protease biochemistry of importance to understand clinical conditions with extensive protease activation.

## Introduction

Proteases are expressed ubiquitously in all tissues of the human body. By definition, the word “protein-ase” derives from the Greek word “protelos” (πρωτελοσ) meaning “first rank” [1] in combination with the word “diastasis” (διάστασις) meaning “cleavage” or “separation.” Proteases capable of cleaving “first-rank” molecules seem to be excessively released and activated under local inflammatory conditions, resulting in local barrier breakdown, cellular dysfunction, and invasion of inflammatory cells and/or microorganisms. There is longstanding evidence that proteases are involved in many, if not all, acute diseases [2].

## General aspects of proteases and enzyme kinetics

Proteases (or proteinases or peptidases) are enzymes that hydrolyze peptide bonds in substrate proteins and are found in all living organisms, including plants. They are classified on the basis of the catalytic residue in the active site of the particular enzyme. The best-studied groups are serine proteases, cysteine proteases, aspartic proteases, and metalloproteases [3], although additional groups have also been defined. The overall reaction mechanism of a protease-mediated cleavage involves a nucleophilic attack on the target peptide bond in the protease substrate. Generation of a nucleophile can involve a catalytic triad of amino acids (as in serine and cysteine proteases), where a histidine residue removes a proton from a hydroxyl or a thiol in the serine or cysteine, respectively. Aspartic proteases and metalloproteases both activate a water molecule, which performs the nucleophilic attack. Metalloproteases have an obligate need for a divalent metal cation in the active site of the enzyme: usually zinc, but in some cases also cobalt.

Here, we present a selection of proteases within each of the main groups that are relevant to the scope of this review article [3]. Within the serine protease group, the chymotrypsin family includes numerous proteases within the complement system, the contact activation (kallikrein)/coagulation system, and the fibrinolytic system. This family also includes enzymes derived from the pancreas such as elastases, endopeptidases, trypsins, and chymotrypsins; leukocyte-originating proteases such as cathepsin G, proteinase 3, and leukocyte elastase; and various tryptases and granzymes. Relevant enzymes within the cysteine protease group are papain, cathepsins (B, H, K, L, S), and calpains, and those within the aspartic protease group are pepsin and gastricsin (or pepsinogen C), renin, and cathepsins D and E. The metalloprotease group includes matrix proteases such as collagenases and gelatinases, which are involved in tissue remodeling, as well as enzymes within the ADAMTS family. In addition, various carboxypeptidases (exopeptidases) belonging to this group are released into the plasma, where some can desarginate and reduce the activity of bradykinin and the anaphylatoxins C3a and C5a. A summary of proteases relevant to this review article and examples of substrates within the complement system is given in Table 1.

**Table 1:** Selection of proteases within the each of the main groups (serine proteases, cystein proteases, aspartic proteases, and metalloproteases), which have been reported to cleave complement components. Proteases within the complement system, which all belong to the serine protease group, (C1r, C1s, MASP1, MASP-2, MASP-3, C2, factor B, factor D, and factor I) are not included in the table

	Complement substrate	Subtype	Reference
Serine proteases			
Trypsins	C1q		[45]
	C1r		[51]
	C4		[53, 56]
	C3		[17, 24, 26]
	C2, Factor B		[54, 55]
	C5		[36]
Chymotrypsins	C1q		[45]
	C3		[24, 25]
Kallikreins	C1s	Plasma	[50]
	iC3b	Plasma	[20]
	iC3b, C5	KLK3 (PSA)	[22]
	C3	KLK14	[23]
Elastases (leukocyte pancreatic)	C3	Leukocyte	[27, 28]
Cathepsin A, G	C3	G	[154]
Chymase	C3		[155]
Tryptase	C3, C5, C4	Beta	[156]
Granzyme A, B, C, D, E, F, G, Y	C3, C5	B	[72]
Coagulation/contact system proteases			
FXIIa	C1r, C1s		[52]
FIXa, FXIa	C5, C3		[34]
FXa	C5, C3		[33, 34]
Thrombin	C5		[32, 35]
	C3, C5		[33, 34]
Fibrinolysis system proteases			
Plasmin	C1s		[49]
	C3, C5		[33, 34]
FSAP (factor VII-activating protease)	C3, C5		[67]
Cysteine proteases			
Papain	C1q		[44]
Aspartic proteases			
Pepsin	C1q		[44, 46]
Cathepsin D, E	C5	D	[82]
Metallo- proteases			
Collagenases (neutrophil, interstitial)	C1q		[45, 47, 48]
Carboxypeptidase A, B, H, M, N	C3a, C5a	B, N	[41, 43]

The specificity of a given enzyme for a substrate varies greatly: promiscuous proteases like trypsin recognize single basic amino acids (Arg or Lys) on the substrate and are consequently able to cleave any protein containing and exposing either of these two amino acids [4]. Like trypsin, thrombin also cleaves its substrates on the carboxyl side of Arg and has multiple substrates, but unlike trypsin, it cleaves each protein at specific positions and with defined kinetics [5]. Elastases usually cleave proteins on the carboxyl-terminal side of amino acids with small, uncharged groups, such as Ala, Gly, or Val [4].

The likelihood for a protease to cleave a given protein target may be expressed in terms of enzyme kinetics as *k*_cat_/*K*_m_, or the catalytic efficiency of the enzyme. For an enzyme that follows Michaelis-Menten kinetics, *V*_max_ is the maximal velocity at saturating substrate concentration, *K*_m_ is the substrate concentration at which the reaction rate is half of that at *V*_max_, and *k*_cat_ is the turnover number, which relates *V*_max_ to the concentration of active sites on the enzyme [6]. *k*_cat_/*K*_m_ can be used as a measure of the substrate preference or the catalytic efficiency of the enzyme, or both.

Chymotrypsin is the archetype serine protease, being able to cleave proteins on the carboxyl side of a large hydrophobic or aromatic amino acid. Its substrate preference has been demonstrated using a series of substrates, with increasingly hydrophobic amino acids giving higher and higher *k*_cat_/*K*_m_ values, indicating that the most efficient cleavage is obtained for the aromatic amino acid Phe [6].

## Properties and specificities of the main intravascular protease inhibitors

Proteases can be generated in the blood by the activation of the plasma cascade systems (the complement system, the coagulation/contact/kallikrein system, or the fibrinolytic system), which in most cases, lead to the activation of zymogen molecules to yield active proteolytic enzymes. Alternatively, proteases may be released from activated leukocytes and/or damaged tissue or invading pathogens, as in sepsis. Regardless of their origin, proteases present in the blood are controlled by a number of protease inhibitors, which make up 10% of the total amount of plasma proteins [7]. The inhibitors show great differences in specificity and concentration. C1 inhibitor (C1-INH) and antithrombin (AT) inhibit the recognition proteases of the classical and lectin pathways of complement and the proteases, which are activated within the contact system. These are factor(F)XIIa, FXIa, kallikrein, and plasmin. AT also inhibits FVIIa, FIXa, FXa, and thrombin within the coagulation system, in addition to trypsin. The serine protease inhibitors (serpins) α1-antichymotrypsin, α1-antitrypsin (which has the alternative designation α1-proteinase inhibitor), α2-antiplasmin, and inter-α-trypsin inhibitor mainly inhibit other types of serine proteases (i.e., in addition to those generated within the complement and coagulation systems) such as cathepsin G, chymotrypsin, plasmin, and trypsin in different combinations (α1-antitrypsin and also collagenases) [8]. Finally, α2-macroglobulin shows the broadest specificity and can inhibit almost any protease of any class, including those of pathogenic origin [9]. A summary of the target specificities and plasma concentrations of these proteases is given in Table 2.

**Table 2:** Main protease inhibitors in human plasma. Normal concentration and targets

Inhibitor	Mw	Plasma concentration		Targets (preferred target first)
	kDa	mg/L	μM	
C1-inhibitor (C1-INH)	100	240	2.4	C1r, C1s, MASP-1/2, FXIIa, FXIa, kallikrein, plasmin
Antithrombin (AT)	58	240	4.1	Thrombin, FXa, C1s, MASP-1/2, FXIIa, FXIa, kallikrein, VIIa, FIXa, plasmin, trypsin
α1-Antichymotrypsin	63	490	8	Cathepsin G, chymase, chymotrypsin
α1-Antitrypsin (= α1 proteinase inhibitor)	53	2900	55	Elastase, trypsin, chymotrypsin, collagenases, cathepsin G, plasmin
α2-Antiplasmin	68	70	1	Plasmin, trypsin, (chymotrypsin, kallikrein, FXa, FXIa)
Inter-α-trypsin inhibitor	180	500	2.8	Trypsin, chymotrypsin, plasmin (slow)
α2-Macroglobulin	725	2600	3.6	Most proteases of all classes including cathepsins, granulocyte and pancreas proteases, collagenases, kallikrein, FXa, thrombin, and plasmin

## Proteolytic cleavage of key complement components by complement and non-complement proteases

### Complement component C3

**Fig. 1:**
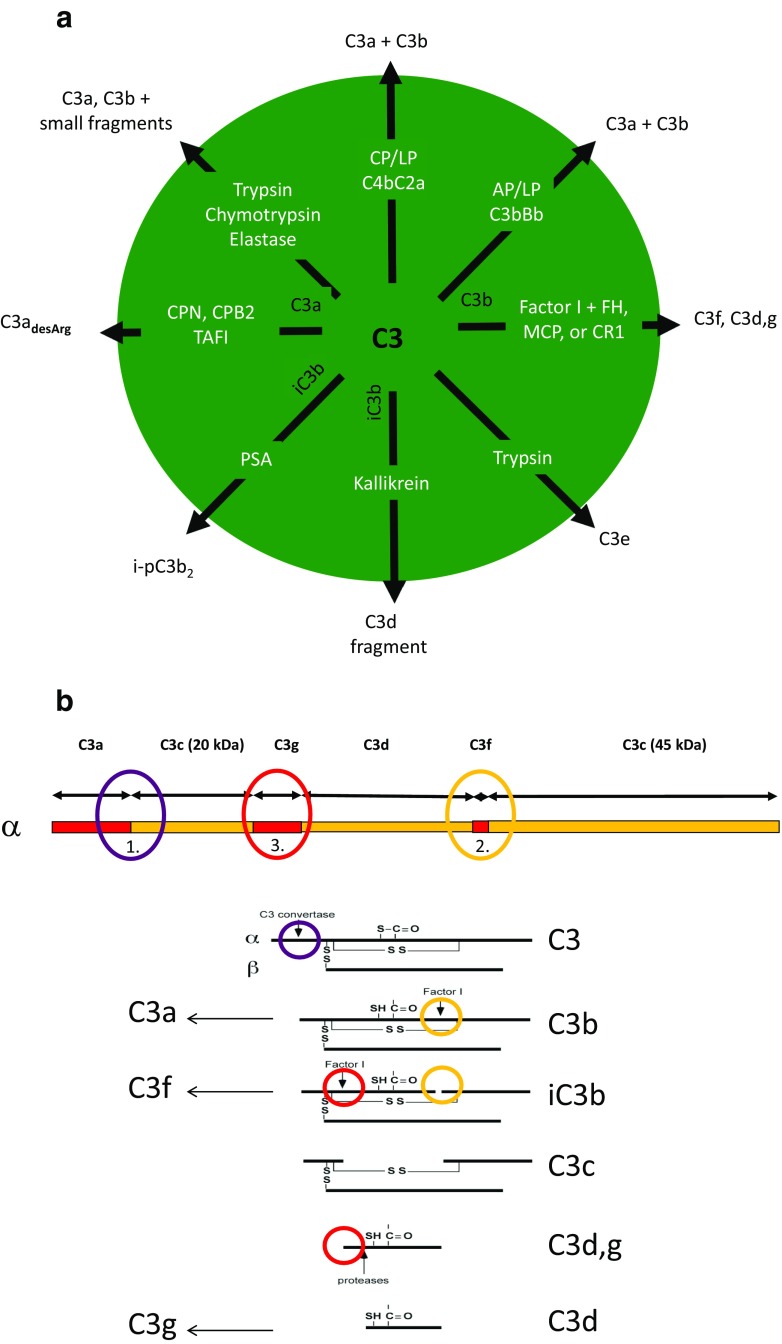
Cleavage of C3 by complement and non-complement proteases. **a** Summary of cleavages; **b** localization of reported cleavage sites in three regions in the α-chain of C3 (top) leading to generation of C3-fragments. C3 is activated to C3a and C3b, by the C3 convertases of the classical/lectin pathways and/or the alternative pathway (1). C3b is subsequently cleaved by factor I together with co-factors into iC3b (2), C3c, C3d,g, and smaller fragments (3). Similar cleavages can also be accomplished by proteases like trypsin, chymotrypsin, and elastase. In addition, iC3b has been reported to be cleaved by PSA (KLK3) to i-pC3b_2_ (2) and by kallikrein (KLK1B) to C3d-k (3). C3a is cleaved at the C-terminal end by carboxypeptidases generating C3a_desArg_ (1). *AP* alternative pathway, *CP* classical pathway, *LP* lectin pathway, *PSA* prostate-specific antigen, *FH* factor H, *MCP* membrane cofactor protein, *CR1* complement receptor-1, *PSA* prostate-specific antigen, *KLK* kallikrein, *CPN*/*B2* carboxypeptidase N/B2, *TAFI* thrombin-activatable fibrinolysis inhibitor

The proteases that cleave the α-chain of C3 under physiological conditions split the protein at distinctive sites within the molecule to yield characteristic fragments (Fig. 1a). These sites are to some extent shared with non-complement proteases, which generate activation products similar to the physiological fragments. It is therefore appropriate to start by describing the physiological fragmentation of C3 that occurs during C3 activation. The initial proteolytic cleavage of C3 is made by the classical/lectin and the alternative pathway C3 convertases (C4bC2a and C3bBb, respectively), which are both restricted to cleaving one Arg-Ser bond at residues 726–727 in the C3 α-chain [10] to produce C3a (which is released) and the larger C3b molecule [11] (Fig. 1b).

The next three physiological cleavages are mediated by the serine protease factor I. Factor I exposes its catalytic site directly upon contact with C3b [12], but in order for cleavage to occur, there is an absolute requirement for one of several co-factors: factor H in the plasma, or membrane-bound MCP or CR1 [13, 14]. Its cleavage sites are located close together in the CUB domain of C3b. The first is located at positions 1281–1282, and the second at positions 1289–1290; both are Arg-Ser sequences whose cleavages produce C3f and the main iC3b fragment [15, 16] (Fig. 1b). A third major cleavage occurs at positions 932–933 (Arg-Glu) [17], cleaving the C3d,g fragment from iC3b to produce the larger C3c fragment (Fig. 1b). An additional cleavage at 937–938 (Lys-Glu) has also been reported [12].

C3a binds to C3aR and C3b interacts with complement receptor 1 (CR1, CD35), iC3b binds to CR3, and CR4 (CD11b/CD18; CD11c/CD18) binds to CR2 (CD21), and C3d,g is another ligand for CR2. The differential binding of the C3 fragments represent regulation of C3 function. From being a fragment for cell lysis, cell adherence and cell activation (CR1, CR3, and CR4) during phagocytosis, cytotoxicity, etc., C3 becomes transformed to a ligand for immunoregulation (CR2), thereby linking innate and adaptive immunity, reviewed in [18].

Additional physiological fragments have also been described. C3e was first identified as a leukocytosis-inducing peptide, although the origin of this fragment was not identified at the time [19]. A few decades after the identification of the C3e fragment, another possibly related fragment was identified. It was a C3d,g-like fragment that could be generated by cleavage of iC3b using the contact system protease kallikrein (Fig. 1b). This C3d-k fragment contained a few amino acid residues more than C3d,g and exhibited leukocytosis-like properties, possibly linking C3e and C3d-k [20, 21].

Intriguingly, another member of the kallikrein (KLK) serine protease family, KLK3 or prostate-specific antigen (PSA), has been shown to digest purified iC3b (but not C3 or C3b), in addition to C5 (but not C4). The cleavage of iC3b occurs at a chymotrypsin-like cleavage site (Tyr-1348) without the aid of factor H or CR1 and gives rise to a novel fragment originating from the 45-kDa portion (Fig. 1b). The same pattern of C3 cleavage has also been seen in prostatic fluid and seminal plasma, where C3, but not C5, is present [22]. In addition, KLK14 has been reported to cleave C3, thereby generating functionally active C3a without downstream generation of C5a [23].

Trypsin, chymotrypsin, and elastase [4] have broad specificities and cleave the whole native C3 molecule into small proteolytic fragments in a dose-dependent manner [24, 25]. Basically, the cleavage regions are the same as for the physiological cleavages, potentially generating proteolytic fragments with biological activity. For instance, low concentrations of trypsin generate C3a and C3b and facilitate the cleavage of C3d,g to C3d and C3g [17, 24, 26], and elastase has been reported to promote similar digestion [27, 28]. A schematic overview of proteolytic digestion of C3 and the resulting fragments is presented in Fig. 1b.

### Complement component C5

The physiological cleavage of C5, which is homologous to C3, is much less well understood and probably more complex. As with C3, C5 is cleaved into C5a and C5b by C5 convertases, generated by the classical/lectin and alternative pathways [29–31]. In addition, a number of studies have shown that non-complement proteases, particularly proteases from the coagulation cascade (e.g., thrombin, FXa, and plasmin), are able to cleave native C5 into C5a and C5b [32–34] (also discussed below). These findings are contradicted by other studies in which, for example, thrombin has been reported to generate a form of C5, which is cleaved at the corresponding third “factor I cleavage site” and not at the convertase site, to generate a form designated C5_T,_ which is still able to support the formation of the terminal pathway complex, C5b-9 [35]. This form of C5_T_ is also generated when native C5 is exposed to trypsin at low concentrations [36] (Prof. Ulf Nilsson, personal communication). Plasmin and FXa are the proteases that most likely cleave C5 at the convertase site at reasonable concentrations to generate C5a and C5b. The discrepancies between studies may be explained by findings in the C5 crystal structure at a resolution of 3.1 Å [37]. Unlike C3, the cleavage site for the convertases in C5 is hidden in the native molecule, necessitating a conformational change in the molecule before the site becomes accessible. The mechanism inducing this conformational change is not known but is probably the key to understanding the function of the abovementioned proteases and C5 convertases.

### Cleavage of C3 and C5 by coagulation proteases

In addition to these proteases, other activated coagulation factors have been shown to generate C3a and C3b, thereby producing anaphylactic activity. Several components have been reported to have this activity in purified in vitro systems: FXa, thrombin, FIXa, and FXIa in decreasing order of strength [33], in addition to plasmin [34]. Also, complement activation is triggered when FXa in relatively low concentration (> 0.2 μg/mL) is added to serum (Table 3). The activation results in C3a and C5a generation, decreased hemolytic activity, and increased anaphylactic activity. The complement-activating activity is blocked by anticoagulants, e.g., fondaparinux [33]. These studies show that coagulation factors can activate complement in a complex serum milieu. However, it is likely that the activity would be lower in plasma containing intact native coagulation factors, which are the preferred substrates. In many experiments with purified components, the concentrations of proteases added have been close to the theoretical limit, i.e., if the total amount of each zymogen had been converted to an active enzyme. In addition, in these studies, the proteases have been tested one at a time, so the question of possible synergistic effects has not been addressed [33, 34]. There are, however, several situations in the clinical setting in which these conditions are approached, for instance in disseminated intravascular coagulopathy (DIC) or sepsis, when all these systems are exhausted, and the intravascular protease inhibitors have been consumed (e.g., [38, 39]). Furthermore, under such dysregulated circumstances, a small amount of C3 activation (generated by coagulation proteases, alone or in combination) may be sufficient to initiate a full-blown inflammation as a result of amplification by the alternative pathway of complement.

**Table 3:** Normal plasma concentration of zymogens of coagulation proteases reported to cleave complement components (top section). Normal plasma concentration of complement components reported to be cleaved by coagulation proteases (lower section)

Coagulation zymogen	Mw	Plasma concentration	
	kDa	mg/L	μM
FXII	100	30	0.3
FXI	160	5	0.03
FX	55	10	0.2
FIX	55	5	0.09
Prothrombin	72	140	1.9
Plasminogen	90	130	1.4
Complement component	Mw	Plasma concentration	
	kDa	mg/L	μM
C1q	400	250	0.6
C1r	80	50	0.6
C1s	85	50	0.6
C4	200	400	2
C3	185	1500	8
C2	108	20	0.2
Factor B	93	200	2.1
C5	180	80	0.4

A number of human pathogens, e.g., *Borrelia*, *Streptoccocus*, and *Leptospira interrogans* acquire plasminogen on their surfaces and use the proteolytically active plasmin to cleave and inactivate C3 and C5 in order to disarm the host immune defense (see, e.g., [40] and references therein).

### Desargination of C3a and C5a

Both C3a and C5a are cleaved at the C-terminal end by carboxypeptidases, generating C3a_desArg_ and C5a_desArg_ [41]. These fragments have a lower affinity for the C3aR and C5aR1 receptors and are therefore considered inactivated forms of the anaphylatoxins, although they are not totally without effect [42]. The active carboxypeptidases are carboxypeptidase N and carboxypeptidase B2, the latter being more active and derived from procarboxypeptidase R (thrombin-activatable fibrinolysis inactivator (TAFI) of the coagulation/fibrinolytic cascade), further illustrating the linkage between the coagulation and complement systems [43].

## Cleavage of classical pathway components by non-complement proteases

Proteolytic digestion is not a feature of C1q activation, but there are several reports suggesting that C1q may be cleaved by proteases that do not belong to the complement system. Human C1q has shown to be sensitive to digestion by trypsin, chymotrypsin, pepsin, and collagenase [44–47]. In contrast to trypsin and chymotrypsin, collagenase treatment of C1q leads to the production of well-defined fragments, which show only moderately decreased binding to immune complexes when compared to intact C1q [45, 48]. Not surprisingly, C3 and C5 (in addition to the rest of the terminal components) have been shown to be refractory to collagenase [47].

Rabbit C1s has been shown to be activated by plasmin [49]. In addition, it has been demonstrated that kallikrein digestion of activated C1s markedly decreases its hydrolytic activity toward C4 without affecting its activity toward C2 [50].

C1r has been reported to be cleaved by trypsin, but this cleavage occurs at a site that is distinct from the auto-cleavage site and does not activate C1r [51]. In addition, C1r and C1s are both activated by FXIIa, and since activation of C1s is much less efficient, it was concluded that it is primarily activated by C1r and only to a lesser degree by FXIIa [52].

C4 and C2 (as well as factor B) are cleaved by trypsin [53–55]. Recently, it was demonstrated by co-crystallization of C4-MASP2 and C4-trypsin, in conjunction with activation measurements, that trypsin indeed is able to activate C4 [56].

## Clinical settings enabling massive release of proteolytic enzymes

In the clinical setting of acute pancreatitis or during the development of abscesses, inflamed/infected tissues may be decomposed as a result of uncontrolled activation of proteases. Moreover, in systemic inflammatory conditions, proteases seem to act as major drivers of pathophysiological inflammation and organ dysfunction. For example, during sepsis, defined as a systemic inflammatory response syndrome (SIRS) in the presence of pathogens, enhanced levels of trypsin [57], elastase [58], and metalloproteinase 9 [59], as well as many other enzymes, are detected and are held responsible for the generation of the inflammatory response. It is noteworthy that the released proteases can originate from the host and/or from invading microorganisms that contain enzymes with similar or diverse functions. For example, in the case of Lyell’s syndrome, manifested as an acute life-threatening epidermolysis, two different forms exist: one is a sterile, drug-induced toxic epidermal necrolysis (TEN), in which host proteases are responsible for detaching the epidermis from the dermis after drug administration, making the entire body susceptible to subsequent infectious invasion and complications. The other form, known as staphylococcal scalded skin syndrome (SSSS), is induced by the serine protease exfoliatin, which is released by invading *Staphylococcus aureus* and finally results in a highly specific breakdown of desmosomes [60, 61].

**Fig. 2:**
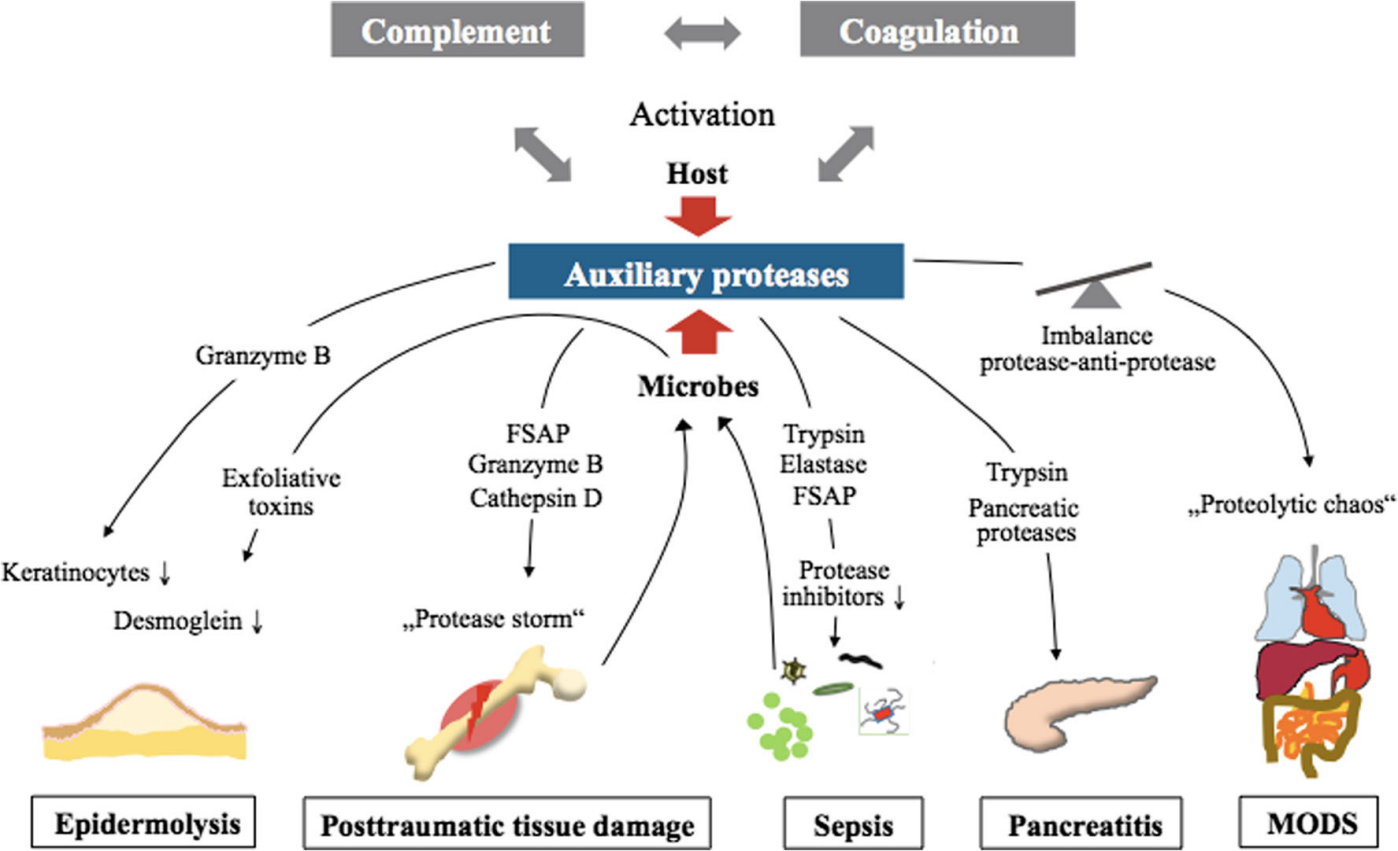
Involvement of protease activity in various diseases. Activation of the interconnected host complement and coagulation cascade can augment cleavage of auxiliary proteases and vice versa, inducing the release of downstream effectors responsible for disease induction or generalization. Proteases released from damaged tissue after trauma or invading pathogens during sepsis can further increase the inflammatory response. *FSAP* factor VII-activating protease, *MODS* multiple-organ dysfunction syndrome

In other diseases associated with complex systemic reactions, such as hemorrhagic shock (HS), acute kidney injury (AKI), and adult respiratory distress syndrome (ARDS), proteases function as important inflammatory trailblazers. Excessive systemic activation of proteases may even result in a proposed “autodigestion” phenomenon [62, 63], which may be clinically crucial for the development of multiple-organ dysfunction syndrome (MODS) and often fatal multiple organ failure (MOF). In the case of multiple trauma (polytrauma), protease release and activation have been described [64–67] and may also be responsible for systemic activation and depletion of the coagulation system, manifested as acute trauma-induced coagulopathy. On the other hand, the intensive crosstalk between the complement and coagulation cascades, accompanied by platelet activation and various proteases and their inhibitors, is critically involved in any thrombus formation [68]. Taken together, these findings indicate that in many acute diseases, proteases can act as local and systemic groundbreakers for tissue invasion by pathogen-associated molecules (PAMPs) and immune cells, generating all the classical signs of inflammation (“tumor, rubor, dolor, calor, function laesa”), which can easily affect the whole body. Therefore, specific therapeutic targeting of proteases beyond the complement and coagulation cascades represents a promising subject for research and clinical investigations in the future [69]. An overview of different clinical settings, which enable massive release of proteolytic enzymes is found in Fig. 2.

## Protease crosstalk in various clinical conditions—therapeutic implications

### Acute life-threatening epidermolysis

Several decades ago, both the complement and coagulation systems were proposed to be important actors in the development of TEN (Lyell’s syndrome), which is caused by proteolytic shedding of the epidermis in response to certain drugs or occasionally to microorganisms [70]. Stevens-Johnson-syndrome (SJS) and TEN differ only in their degree of epidermal detachment, with an affected body surface area of < 10% in case of SJS and > 30% for TEN (with SJS/TEN overlap in between). Pathophysiologically, this disease represents a severe hypersensitivity reaction, with activation of cytotoxic T lymphocytes and natural killer cells; these cells, in turn, mediate immune reactions directed at keratinocytes. Furthermore, the immune reaction is associated with the release of cytokines, perforin, granzyme B, and several proteases, finally resulting in keratinocyte death and disintegration of the epidermis [71]. Although it is not yet implicated in this life-threatening skin disease, the serine protease granzyme B has been shown to directly activate complement in a non-canonical manner, generating the anaphylatoxins C3a and C5a [72], which can induce all the cardinal signs of inflammation. Given the rarity of these diseases, there are no randomized clinical trials for SJS or TEN. However, in some clinical case reports, trypsin inhibition by ulinastatin has been beneficial [73].

The related disease SSSS, caused by PAMPs, toxins, and proteases from *Staphylococcus aureus*, seems to critically involve complement activation and dysregulation [74]. *Staphylococcus* and its toxins can not only activate complement and coagulation but can also evade complement surveillance [75]. For example, there are staphylococcal complement inhibitors (SCINs) that effectively stabilize C3 convertases in a non-functional state, allowing them to escape complement attack [75]. Exfoliative toxins from *S. aureus* are serine proteases that cleave desmoglein 1, the most important component for the structural integrity of the epidermis [76]. Whether these toxins can also cleave, activate, or deactivate complement or coagulation factors remains to be clarified.

### Trauma and hemorrhagic shock

After severe tissue injury, a rapid activation of both the complement and coagulation cascades has been reported [77, 78], with the potential development of a harmful trauma-induced coagulopathy and complementopathy [77]. Although the importance of crosstalk between the two cascades has been postulated, only a few clinical studies have assessed its direct impact and importance for traumatized and critically ill patients. One study has analyzed septic patients as compared to multiple-injury patients and has found enhanced plasma levels of C3a, C5a, and leukocytes but reduced plasma fibrinogen and platelet counts [79]. Remarkably, in addition to increased MOF scores and lactate concentrations, reduced C1-INH activity was the only reliable predictor of poor outcome [79]. This result emphasizes the importance of cross-regulatory drivers, since C1-INH blocks not only the activation of complement and coagulation but also the activation of the contact system. It is tempting to speculate that after polytrauma, extensive tissue damage, ischemic stress, acidosis, hypothermia, and reactive oxygen radicals may locally generate complement and coagulation activation products in an attempt to control bleeding and inflammation. Furthermore, systemic activation of protease systems beyond the complement and coagulation cascades may result in a “proteolytic chaos,” which in turn will further (non)specifically cleave and activate complement and coagulation factors. It is still unknown whether these cleavage products appear as native proteins with established structure-function relationships or exhibit novel functions. Extensively enhanced activity factor VII-activating protease (FSAP), representing an ancient coagulation factor, was found to be associated with increased plasma levels of the anaphylatoxins C3a and C5a early after polytrauma [67]. In vitro exposure of FSAP to key complement components revealed its ability to cleave C5 into a C5a fragment, which, however, lacked four amino acids at the N terminus, interestingly without altering the molecule’s functional profile. FSAP-induced C3 cleavage resulted in two differently sized cleavage products, whose exact functions in vitro and in the clinical setting are still unknown [67].

After clinical trauma to the thorax, enhanced levels of C5a have been found in bronchoalveolar lavage (BAL) fluids, depending on the volume of the contused lung tissue and subsequent development of pneumonia [80]. In the same study, the authors showed enhanced C5a levels in BAL fluids after trauma in a re-translational approach employing a rodent thorax trauma model. Interestingly, blockade of thrombin, a potent serine protease, by hirudin not only reduced thrombin levels but also significantly reduced C5a concentrations, pointing to thrombin-induced complement activation as a major contributor to acute lung injury after blunt chest trauma [80]. Similarly, plasma concentrations of the serine protease granzyme B were shown by our group to be increased in the early stages after multiple injuries in humans and again to be able to generate anaphylatoxins in vitro [72]. Moreover, granzyme B has also been shown to cleave von Willebrand factor (vWF) and the matrix form of fibrinogen at several sites, resulting in an overall anticoagulatory activity [81]. Other protease species seem to be generated rapidly after severe tissue trauma. For example, the aspartyl-protease cathepsin D is significantly increased in plasma from patients early after polytrauma and may generate a C5a-like protein, although cleavage of the classical serine moiety is rather unlikely [82].

The exact origins and mechanisms of protease activity after severe tissue trauma are not well defined. Nevertheless, it is somehow surprising that some protease inhibitors that not only effectively address protease-induced host damage but also control the coagulation, and complement systems have not been tested in the experimental and clinical trauma setting. A previous experimental report in trauma-hemorrhagic shock described improved survival rates when an elastase inhibitor was applied, resulting in reductions in a cardio-depressive factor [83]. Similarly, in a traumatic spinal cord injury model, the elastase inhibitor showed promising results in blocking the sensor system for nitric oxide, promoting functional recovery, and reducing the inflammatory response. However, nothing was reported about complement or clotting effects in particular [84], and no valid translational data are available.

In case of the trypsin inhibitor ulinastatin, a small study in trauma-hemorrhagic shock patients has been performed and revealed only modest early effects, such as reduction in serum neutrophil-derived elastase within 45 h, but no effects on clinically relevant parameters or scores [85]. A recent clinical study (NCT01275976) in the complex setting of polytrauma, in which 200 U/kg C1-INH was infused over 30 min (just before the start of the femoral or pelvic fixation operation), has been terminated, but the final results have not yet been published [86]. The most well-developed and well-evaluated protease inhibitors in the clinic, such as aprotinin (Trasylol®), inhibit serine proteases. A meta-analysis examining orthopedic surgery that is associated with significant tissue trauma has indicated that there are benefits from serine protease blockade: reduced blood loss and need for transfusions, and no enhanced risk for thromboembolic events [87]. Other reports in a pig model of hemorrhagic shock have indicated that local application of the synthetic serine protease inhibitor nafamostat produces some improvement in clinical outcome parameters [88]. However, there are no reliable data available for serine protease inhibitory strategies for complex polytrauma conditions.

Taken together, the data indicate that although there is evidence of a “protease storm” after severe tissue trauma, inhibition of specific proteases in the trauma-hemorrhage situation seems to be of limited help in the complex clinical setting. Such an approach may even further disturb the acute trauma-induced coagulopathy, and thus, further experimental pathophysiological investigation is certainly warranted.

### Sepsis

In human sepsis, defined as an infectious whole-body inflammation, it has been established that during the induction and progression of the systemic response, there is a massive activation of the complement system [89, 90] and the coagulation cascade [91]. This activation may result in immune dysfunction, complementopathy, coagulopathy, and (multiple) organ dysfunction. The importance of the complement-coagulation interplay in the pathophysiological development of sepsis has been demonstrated in non-human primates [92]. In particular, systemic C3 depletion has been found to be associated with the development of coagulopathy (elevated D-dimers), infectious complications, and poor prognosis in septic patients [93]. In progressive sepsis, an uncontrolled systemic protease activity has been reported, with enhanced serum concentrations of trypsin [57] and elastase [58, 94] and serine protease activity, e.g., of FSAP [95]. This “global protease activation” may be mainly responsible for the impaired inhibition of endogenous proteases, e.g., a drop in AT plasma concentrations [96]. Accordingly, AT substitution during severe sepsis in humans has been shown to reduce IL-6 concentrations in serum as surrogate parameter and to also reduce overall mortality [97, 98]. Interestingly, in humans, antithrombin, acting as a specific protease inhibitor, has been found to display antimicrobial activity [99] that might prove supportive in sepsis therapy. Furthermore, in septic trauma patients, enhanced elastase levels seem to contribute significantly to the tissue factor (TF)—TF pathway inhibitor (TFPI) imbalance, which in turn aggravates ARDS development and sustained systemic inflammation [94]. Of note, when administered to patients with abdominal sepsis and ARDS, the selective neutrophil elastase inhibitor sivelestat results in early improvement in oxygenation and ventilator weaning, as well as improved multiple organ function [100]. In this context, experimental data have shown that specific elastase inhibition significantly attenuates not only endotoxin-induced septic acute lung injury but also reduces C5a and IL-6 concentrations in the alveolar space [101].

In rodents, sepsis-induced pro-coagulatory activity and synchronic signs of DIC, as indicated by prolonged activated partial thromboplastin time (APTT), reduced platelet count, thrombin-antithrombin (TAT) complexes, D-dimers, and tissue-plasminogen activator (t-PA), are mostly reversed by C5a blockade, resulting in an improved survival rate [102]. This study illustrates the clinical potency of the crosstalking serine protease systems. It is therefore not too surprising that multiple serine protease inhibitory strategies have been clinically applied in the past, albeit not predominantly in sepsis. In a small retrospective single-center study that investigated patients with sepsis-induced DIC, gabexate mesilate as serine protease inhibitor versus recombinant thrombomodulin, thrombomodulin was found to be more effective in improving platelet counts, C-reactive protein (CRP) concentrations, and MOF [103]. At the same time, ulinastatin as a multi-functional serine protease inhibitor has been proposed as “an exciting candidate” for sepsis therapy [104]. Recent meta-analyses have stated that ulinastatin alone or in combination with thymosin α1 indeed results in an improved overall survival, reduced generation of inflammatory mediators, and a shortened need for mechanical ventilation [105–107].

An illuminating study in humans admitted to the intensive care unit with SIRS has revealed that approximately 25% of all proteins mapped to either the coagulation or complement system are differently expressed in patients who develop sepsis versus non-septic SIRS [108]. Strikingly, differences in some complement and coagulation proteins in these SIRS patients were capable of predicting a septic course even 60 h before sepsis became clinically evident. Moreover, 36 h before sepsis onset, the plasma levels of various endogenous protease inhibitors, such as α1-antitrypsin and AT, were decreased, suggesting that specific protease inhibitor application might serve as a reasonable therapeutic strategy for ameliorating or inhibiting sepsis development [108]. However, more clinical studies are needed to precisely define the most appropriate indications, timing, and dosage for protease inhibitors in coagulatory and inflammatory disorders during sepsis.

### Multiple-organ dysfunction syndrome

Multiple-organ dysfunction syndrome (MODS) may be considered a final pathophysiological escalation route in various diseases, such as polytrauma, severe infection, sepsis, and hemato-oncological diseases. MODS, defined by a significant incremental functional derangement of at least two or more organs, is still associated with a high mortality rate. Interestingly, during MODS, the major functional defects are not necessarily reflected by major morphological defects [109], even though multiple functionally crosstalking organ systems such as the liver-kidney, lung-kidney, and brain-gut axes are involved in the complex organ response. In the case of severe ARDS, a clinical proteomic analysis has revealed not only protease-antiprotease imbalances with significantly enhanced truncated forms of α1-antitrypsin but also an associated complement activation with increased C4 levels that is indicative of a tightly interacting pulmonary protease-inflammation response [110]. In this context, it is not too surprising that also in the case of ARDS, a recent meta-analysis of clinical studies has pointed to beneficial effects from blocking trypsin using ulinastatin, although data on complement and coagulation were missing [111]. In the last two decades, crosstalking complement and coagulation cascades have also been described as being involved in triggering and promoting the development of MODS [112–115]. Consequently, blockade of C5a in cecal ligation and puncture-induced sepsis and subsequent MODS has proved protective not only with regard to the coagulatory response [102] but also for organ performance, i.e., improved oxygenation of the lungs, reduction in lactate generation, and amelioration of acute liver and kidney injury [113]. In a non-human primate model of *E. coli*-induced MODS, blockade of complement factor C3 by the inhibitor compstatin has not only inhibited central complement activation but also reduced signs of coagulopathy (reduced TF, plasminogen activator inhibitor-1 (PAI-1), fibrinogen, fibrin-degradation products, APTT) while maintaining the anticoagulatory properties of the endothelium [116]. Furthermore, compstatin has improved the hemodynamics and, concurrently, liver and kidney function, with evidence of fewer microvascular thrombi and improved vascular barrier function [116]. In the clinical setting, an integrated clinico-transcriptomic approach has recently correlated early thrombocytopenia with MODS development and C5 expression levels with nosocomial infections [117].

Regarding the therapeutic inhibition of non-canonical crosstalking protease systems, the trypsin inhibitor ulinastatin has been found to exert some protective effects on the immune response and hemostasis [118]. Similarly, in vitro thrombelastometric analyses have indicated some anticoagulatory effects [119]. Translational studies in abdominal surgery have shown evidence not only of anti-inflammatory effects (via inhibition of neutrophil elastase activity) but also of anticoagulatory and antifibrinolytic effects (via reduction in the levels of TAT complexes, plasmin-α2antiplasmin inhibitor-complexes (PIC), and fibrin/fibrinogen degradation products [120]). In another study in severe sepsis patients, ulinastatin has impressively reduced the incidence of MODS onset by more than 50% [121]. Furthermore, another serine protease inhibitor, gabexate mesilate, has shown promising results in a rodent endotoxin-induced MODS model [122]. Short-term pretreatment has resulted in improved lung, liver, and kidney function; a reduction in both consumptive coagulopathy (determined by thrombelastography) and the inflammatory response; and a reduction in TAT and PAI-1 generation.

Temporary artificial organ replacement or assistance devices to bridge functional organ derangement during full-blown MODS may themselves activate both the complement and coagulation systems, as has been shown in the case of an extracorporeal liver-assist device [123] and lung-assist device [124]. The latest improved extra-corporeal oxygenation systems still exhibit a tendency for C3 consumption and TAT complex formation after a 6-h running time [125]. On the other hand, surface coating of an extracorporeal membrane oxygenation device with nafamostat mesilate has recently led to an increased bleeding risk when compared to standard heparin use [126]. Thus, molecular surface engineering is required for future organ assistance systems and their artificial surfaces in order to prevent cross-activation of the complement and clotting cascades [127]. Furthermore, bedside monitoring of protease activity, including the complement and coagulation systems, is desirable to specifically target and stop the vicious cycle of protease activation and subsequent immune, coagulation, and organ failure.

### Pancreatitis

Although the role of the clotting cascade in the development of acute pancreatitis is well established, the function of complement in this process and the likely crosstalk of both systems, triggered by massive protease release, remain unclear, as indicated in a recent review [128]. In acute pancreatitis, enhanced plasma levels of C3a and the terminal complement complex (sC5b-9) have been found in humans and are correlated with the severity of the disease [129]. Nevertheless, in the case of acute pancreatitis, pancreas-derived proteases such as trypsin have been proposed to function as the culprit in MODS development [130]. Supporting data has come from a meta-analysis showing that ulinastatin can improve indicators of inflammation in acute pancreatitis [131]. Another meta-analysis has shown nafamostat, with its strong coagulation and complement inhibitory profile [132, 133] to be effective in post-endoscopic retrograde cholangiopancreatography (ERCP)-induced pancreatitis [134, 135].

In the past, experimental data in acute hemorrhagic pancreatitis in sheep have indicated that the serine protease inhibitor aprotinin (Trasylol®) significantly reduces the pathophysiological consequences of crosstalking systems, such as DIC, granulocyte sequestration, and remote lung vascular permeability [136]. However, translational studies have revealed less convincing results, and therefore, the application of aprotinin has been abandoned for treatment of pancreatitis, especially since the hyperfibrinolytic state in pancreatitis, as major target of the aprotinin therapy, could not be monitored reliably in the pre-thrombelastography era. Currently, aprotinin administration is mainly restricted to complex cardiac surgery (see below) and major orthopedic surgery, where it has been shown to reduce transfusion requirements without increasing the risk of thrombosis [87]. Although mechanistically rational, protease inhibition during pancreatitis to improve clotting and inflammatory complications would benefit greatly from protease monitoring, which is not yet on the horizon.

### Cardiac surgery involving cardiopulmonary bypass

Major cardiac surgery is known to induce a certain degree of systemic inflammation, with cross-activation of the coagulation and complement system, particularly under on-pump conditions [137–141]. Activation of the kallikrein-kinin-system, bradykinin, factor XIIa, and t-PA by cardiopulmonary bypass (CPB) surgery, along with enhanced levels of systemic inflammatory cytokines such as IL-6, IL-8, and TNF, is well established [139]. Simulated extracorporal circulation experiments have suggested that both the contact and complement systems are activated (as evidenced by the formation of C1-C1-INH and kallikrein-C1-INH complexes, as well as C5a-dependent neutrophil elastase generation), all of which can be inhibited by a specific kallikrein blocker [142]. However, in its translation to the clinic, inhibition of the serine protease kallikrein is achieved, for example, by administration of full-dose aprotinin, which is thought to have antifibrinolytic and anti-inflammatory characteristics. This therapeutic approach around CPB operations remains questionable, given the data from single-center studies and also from meta-analyses [143–145]. Several other protease systems, such as cathepsin D [146] and trypsin [147], have been reported to be activated during CPB, and these systems may, in turn, directly cleave key components of the complement cascade [82]. Therefore, trypsin inhibition by ulinastatin had originally seemed a rational therapeutic approach. However, based on a recent meta-analysis of randomized clinical trials, ulinastatin has failed to significantly modulate the cytokine profile but has reduced the duration of mechanical ventilation clinically required [148]. In contrast, another meta-analysis that included CPB patients in China and Japan indicated that ulinastatin not only decreased the need for mechanical ventilation but also improved cytokine profiles [149]. Another therapeutic strategy for inhibiting crosstalking inflammation and coagulation has been to address the potent serine protease FXIIa, which bridges the intrinsic pathway of the clotting system and the classical pathway of the complement system. By inhibiting FXIIa, the antibody 3F7 has blocked not only FXIa generation and formation of C3 activation products but also thrombin, fibrin, and kallikrein activation [141]. In conclusion, 3F7 seems to be equal in potency to the anticoagulatory effects induced by heparin and has therefore been suggested as a safe and promising alternative for heparin in CPB surgery [141].

A critical review of the evidence base for more than 30 different interventions modulating the unquestionable systemic response after CPB has suggested that blockade of C5 and C1r/s has several clinical benefits, including myocardial protection [150]. C1-INH, as a pleiotropic agent targeting all the coagulation, complement, and fibrinolytic systems, has been considered ideal for the “multi-hit” model of CPB [150]. In the case of C5 inhibition, the monoclonal anti-C5 antibody pexelizumab has failed to significantly improve overall survival in CPB patients. However, it conferred a clear survival benefit on a high-risk surgical CPB subgroup throughout the 180-day observation period [151, 152]. Nevertheless, the effects of C5 inhibition on the coagulation system in CPB patients have not been documented and need future detailed consideration. A promising therapeutic strategy for maintaining the terminal complement activation pathway is reflected by a current phase II study in complex cardiac surgery that is using a monoclonal C5a antibody to target the peak IL-6 level as a primary endpoint [151]. Another promising approach in CPB surgery is the modification of the surface coating of the bypass circuit. One study has used a heparin-coated closed-circuit system, which results in lower concentrations of C3a, sC5b-9, t-PA, neutrophil elastase, and IL-8 during the rewarming phase [153]. Overall, coating strategies seem to be essential for modulating the systemic inflammatory response after CPB and may therefore include both the complement and coagulation inhibition that have classically been achieved by heparin coating. Here, specifically addressing the contact system seems to be an interesting clinical goal.

## Conclusion

Under physiological conditions, activation of the cascade systems is under strict control as a result of the high specificity of the generated enzymes and the influence of obligate co-factors, which limit the risk of cross-activation of the systems. However, there are many clinical settings that feature massive release of proteolytic enzymes with auxiliary functions that allow them to circumvent this regulation and enable uncontrolled complement-coagulation crosstalk. Furthermore, the presence of such enzymes seems to be of central pathophysiological significance in several diseases. For successful clinical studies, more research efforts are needed to define the critical extent of the protease activity and the exact mechanisms responsible for disease generation and deleterious outcomes. Still, in order to avoid the risks and adverse effects associated with inhibition of central components of the complement and coagulation cascades, specific and targeted interference with downstream proteases exerting de facto harmful functions may prove to be a valuable tool in preventive therapeutic strategies.
